# Current concepts of tocilizumab efficacy in active moderate-to-severe corticosteroid-resistant cases of Graves’ orbitopathy

**DOI:** 10.3389/fendo.2025.1632531

**Published:** 2025-09-11

**Authors:** Georgios Boutzios, Anastasia Nikolopoulou, Sofia Chatzi, Athanasios G. Tzioufas, Andreas V. Goules

**Affiliations:** ^1^ Department of Pathophysiology, National and Kapodistrian University of Athens, Athens, Greece; ^2^ Research Institute for Systemic Autoimmune Diseases, Athens, Greece; ^3^ Biomedical Research Foundation Academy of Athens, Athens, Greece

**Keywords:** Graves’ orbitopathy, thyroid eye disease, IL-6, tocilizumab, efficacy

## Abstract

Graves’ orbitopathy (GO) is an autoimmune disease affecting the orbit and the retro-ocular tissues. GO pathogenesis involves multiple complex mechanisms, including the contribution of many inflammatory cytokines, such as interleukin-6 (IL-6). GO severity ranges from mild to severe and sight-threatening cases, with the latter affecting only a small percentage of patients. A considerable number of these patients do not respond to first-line immunosuppressive therapy with weekly intravenous pulses of corticosteroids and therefore, there is an unmet need for a second-line treatment, based on immunosuppressive drugs. In recent years tocilizumab (TCZ), an IL-6 inhibitor, has emerged as an effective and safe alternative option for the treatment of active, moderate-to-severe, refractory to steroids cases of GO. This review focuses on the up-to-date concepts regarding TCZ administration for the management of these patients.

## 1 Introduction

Graves’ orbitopathy (GO) is an autoimmune disorder affecting the orbit and the retro-ocular tissues, occurring in 30%-50% of patients with Graves’ disease (GD) (1). GO ranges from mild, non-progressive to moderate and severe cases, clinically expressed as constant diplopia, dysthyroid optic neuropathy (DON) and corneal breakdown. However, only 5-6% of patients experience signs and symptoms of moderate-to-severe disease (2). Of note, moderate-to-severe GO is defined as the presence of at least two of the following clinical findings: lid retraction ≥ 2mm, proptosis ≥3 mm above normal for gender and race, moderate to severe involvement of the soft tissues or/and diplopia which may be inconstant or constant (2). Clinical Activity Score (CAS), although having some limitations, is the most efficient validated scoring system of disease activity (1). It consists of seven components including spontaneous retrobulbar pain, pain on attempted upward or downward gaze, redness of the eyelids and/or of the conjunctiva, swelling of the caruncle or plica, swelling of the eyelids and chemosis, with each one being scored with 1 point. GO is characterized as active when CAS is ≥3/7 (2).

Risk factors for disease development and severity progression in individuals with GD include high serum titer of thyroid-stimulating immunoglobulins (TSIs), radioiodine treatment, thyroid dysfunction (hyperthyroidism or hypothyroidism), tobacco use and hypercholesterolemia (2).

Based on the current European Group on Graves’ Orbitopathy (EUGOGO) guidelines, first-line treatment of active moderate-to-severe GO consists of intravenous corticosteroids (4.5g cumulative dose of methylprednisolone) either as monotherapy or in combination with mycophenolate mofetil (2). However, there are corticosteroid-resistant cases for which appropriate management is the object of ongoing research. Second-line treatments include higher cumulative doses of corticosteroids, other immunosuppressive agents, such as azathioprine and cyclosporine as well as surgical orbital decompression and orbital radiotherapy (2). Currently, attention has been drawn to the potential effectiveness of monoclonal antibodies, such as tocilizumab (TCZ) and teprotumumab.

Tocilizumab (TCZ) is a humanized monoclonal antibody directed against the interleukin (IL)-6 receptor, already approved for use in rheumatoid arthritis and juvenile idiopathic arthritis (3). Given that IL-6 signaling pathway constitutes one of the main pathways involved in the GO pathogenesis, this review aims to summarize the up-to-date knowledge regarding the use of TCZ for active moderate-to-severe GO cases which have not responded to corticosteroids and to determine when the appropriate time is for effective therapeutic intervention with TCZ (4–6).

## 2 Main pathways of pathogenesis involved in GO

The pathogenesis of GO has been studied extensively during the last decades. It has been shown that the thyroid-stimulating-hormone receptor (TSHR), the main autoantigen targeted by autoantibodies in GD, is expressed by orbital tissue (7, 8). Another noteworthy structure is the insulin-like growth factor-1 receptor (IGF-1R), which is expressed by both B cells and T cells in the orbit, as well as by the orbital fibroblasts. IGF-1R is believed to form a functional complex with the TSHR allowing their interaction, that leads to IGF-1R intracellular signaling pathways induction and cytokine production after TSIs binding to TSHR (9).

**Figure 1:**
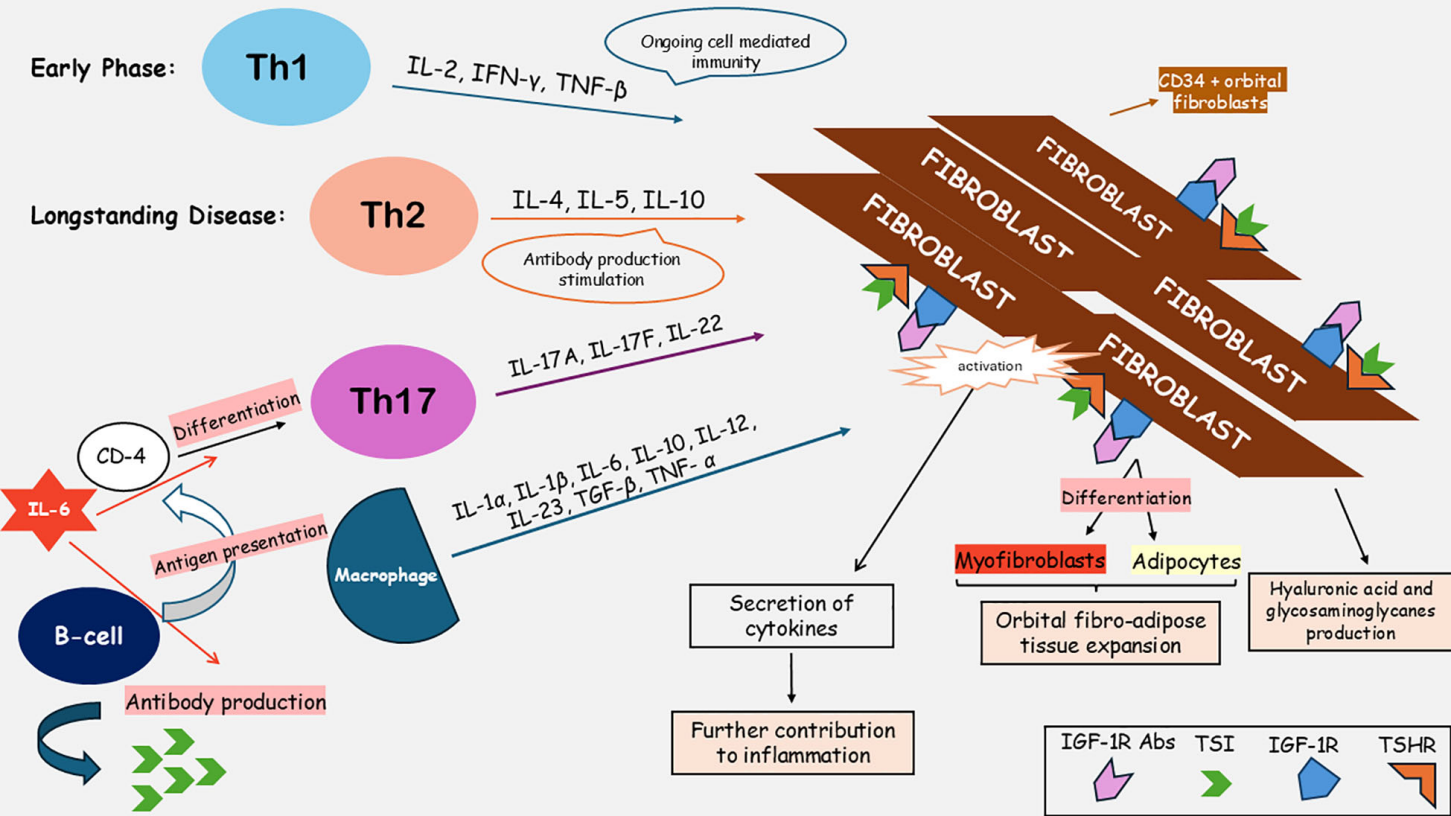
Pathophysiological mechanisms responsible for inflammation and tissue remodeling in Graves’ orbitopathy. IGF-1R Abs, insulin growth factor-1 receptor antibodies; IGF-1R, insulin growth factor-1 receptor; IL-1α, interleukin-1α; IL-1β, interleukin-1β; IL-2, interleukin-2; IL-4, interleukin-4; IL-5, interleukin-5; IL-6, interleukin-6; IL-10, interleukin-10; IL-12, interleukin-12; IL-17A, interleukin-17A; IL-17F, interleukin-17F; IL-22, interleukin-22; IL-23, interleukin-23; IFN-γ, interferon gamma; Th1, type 1 T-helper cells; Th2, type 2 T-helper cells; Th17, type 17 T-helper cells; TNF-α, tumor necrosis factor – alpha; TNF-β, tumor necrosis factor – beta; TGF-β, transforming growth factor-beta; TSI, thyroid-stimulating immunoglobulins; TSHR, thyrotropin-stimulating-hormone receptor.

**Figure 1** summarizes the mechanisms involved in GO pathogenesis. It appears that both cellular and humoral immunity contribute to the inflammatory process in GO. More specifically, B-lymphocytes are responsible for antigen presentation to CD4+ T-cells (10). Type 1 helper T cells, mostly present early in the disease course, produce cytokines such as interleukin 2 (IL-2), interferon γ (IFN-γ) and tumor necrosis factor-beta (TNF-β), which contribute to the ongoing cell-mediated immunity in the orbit. Type 2 helper T cells predominate in long-standing disease, secreting IL-4, IL-5 and IL-10 enhancing autoantibody production. Other inflammatory mediators, such as IL-1β, 6, and 16 and the transforming growth factor-β (TGF-β), are produced within the orbit by several different cell types including macrophages, fibroblasts, and adipocytes (1). Interleukin 6 (IL-6) is among the main cytokines contributing to the orbital inflammatory process in GO. IL-6 promotes the differentiation of CD4 T cells in T-helper 17 (Th17) cells, while it upregulates antibody production by B cells (11). The interleukin 6 receptor (IL-6R) can be found in the surface of target cells as a membrane binding receptor and in soluble form. IL-6 can bind to both forms and activate signaling pathways for gene expression and various other biologic activities. Several studies have attempted to measure the levels of IL-6 in the serum of patients with GD and GO. Molnar and Balazs showed that IL-6 levels were significantly higher in patients with GD and GO than in those with GD but no clinically evident ocular disease (12). Notably, in patients with signs of active inflammation and thyroid disease duration longer than one year, the levels of IL-6 were significantly higher compared to those with recent onset of GD (12).

Fibroblasts constitute the main target cell type of the autoimmune process. Immunohistochemical studies revealed that the CD34+ orbital fibroblasts (OFs) subgroup originating from the bone marrow-derived fibrocytes express TSHR on their surface and after being stimulated by inflammatory cytokines, they further contribute to the inflammation process by secreting pro-inflammatory molecules, such as IL-1β, IL-6 and tumor necrosis factor-alpha (TNF-α) (10, 13–15). Subsequently, they proliferate, synthesize hyaluronic acid, and differentiate into myofibroblasts and adipocytes, resulting in orbital fibro-adipose tissue expansion (15). While it has been shown that molecules, such as IL-17A, IL-23, TNF-α, for which targeted therapies already exist and are being administrated in systemic autoimmune diseases, participate in the pathogenesis of GO, we are currently lacking sufficient evidence to support the use of these treatments in clinical practice for GO (16–19).

## 3 The efficacy and safety of tocilizumab in patients with GO

In this mini-review, we included data from 15 studies that examined the administration of tocilizumab in active, moderate-to-severe, steroid-resistant cases of GO. **Table 1** presents study design, number of patients, patients’ demographics and smoking status, GO activity status and duration, previously administered treatments, TCZ outcome in terms of disease activity improvement, as expressed by CAS reduction and follow-up period duration of the included studies.

**Table 1:** Tocilizumab administration in active, moderate-to-severe steroid-resistant GO – data from previous studies.

Author, year	Study design	Population	Number of patients	Age (years)	Sex (men/women)	Ethnicity	Smoking status – active (yes/no)	GO duration	GO status (Activity)	Previous treatment	CAS before TCZ	CAS after TCZ	Follow up duration
Perrez-Moreiras, 2018 (20)	RCT	Moderate to Severe GO	32	45.07 (median)	8/24	NS	active smokers excluded	1.09 year (median)	Active (10 point CAS >4)	IVMP (32)	5 (Median – TCZ group)	CAS improvement of at least 2 13/15 patients & CAS fall < 3 in 12/15 patients	28 weeks
Sanchez-Bilbao 2020 (21)	Observational	Moderate to Severe GO	48	50.96 ± 11.8 (mean)	10/38	NS	25/23	0,9 years (mean)	Active (7 point CAS >3)	IVMP (43), Selenium (7), Decompressive surgery (7)	4.64 ± 1.5	1.05 ± 1.27	16.05 ± 2.06 months
Perrez-Moreiras,2021 (22)	Retrospective – longitudinal	Moderate to Severe GO	54	53.8 ± 10.5 (mean)	13/41	NS	21/33	10.5 months (median)	Active (10 point CAS >4)	IVMP (54)	6.7 (SD^e^: 1.5)	0.4 (SD: O.7)	22 months
Tobon, 2023 (23)	Retrospective, case -control	Moderate to Severe GO	6	56 (SD: 16)	2/4	Caucasian	1/5	14.5 months (median)	Active (7 point CAS >3)	IVMP (6), Selenium (2), Orbital radiation (2), Decompressive surgery (4)	4	“inactive” 6/6	52 weeks (from therapy initiation)
Boutzios, 2023 (24)	Observational	Moderate to Severe GO	12	58.4 ± 13.4 (mean)	6/6	Caucasian	8/4	2–12 months (mean)	Active (7 point CAS >3)	IVMP (12), MMF (2), Decompressive eye surgery (6)	Median CAS reduction by 3 units (p=0.002)		6 weeks (median)
Moi, 2022 (33)	Retrospective	Moderate to Severe GO	10	At diagnosis: 51 ± 6.2 years (range 40–60)	3/7	NS	6/4	–	Active (7 point CAS >3)	IVMP (10), Orbital radiation (3)	4.80 ± 1.13	0.70 ± 0.82	24 months (12–36 months)
Lee, 2024 (25)	Prospective	Moderate to Severe GO	19	46.4 (mean)	6/13	Asian	4/15	2.1 years (mean)	Active (7 point CAS >3)	IVMP (19), AZA (7), MTX(5), Orbital radiation (13)	4.53	1.68	22.79 months
Bennedjaï, 2022 (34)	Retrospective	Moderate to Severe GO	7	50 ± 12	4/3	NS	3/4	7.97 months (mean)	Active (10 point CAS >4)	IVMP (7), GCs per os (1), Orbital radiation (2), Decompression Surgery (2)	5 ± 0,5	1.2 ± 0.9	11 months (TCZ group)
Pampín-Sánchez, 2023 (26)	Retrospective	Moderate to Severe or Sight-Threatening GO	11	52 ± 12 (mean)	2/9	Caucasian (10), Hispanic (1)	3/8	–	Active (10 point CAS >4)	IVMP (11)	5	1	18 ± 6 months
Jose C-MJ,2020 (27)	Case Series- Descriptive	Moderate to Severe GO	8	–	6/2	Mexican	2/6	–	Active (7 point CAS >3)	IVMP (8), GCs (6), MTX (4), RTX (1), Orbital radiation (2)	4.12± 0.32	1.12 ± 0.61	6 months (after TCZ)
Smith, 2022 (31)	Observational	Moderate to Severe GO	9	55.6 (average age)	0/9	NS	1/8	2.89 (mean)	Active (10 point CAS >4)	GCs	6.78 ± 1.09	0.44 ± 0.53	23.6 months
Wang, 2024 (28)	Retrospective Observational	Moderate to Severe GO	79	39.87 ± 18.50	17/52	Chinese	3/76	18.42 ± 25.18 months (mean)	Active (10 point CAS >4)	IVMP (64), Decompressive surgery (14)	2.10 ± 1.61	1.33 ± 1.49	23.17 ± 21.08 weeks
Copperman, 2019 (29)	Case Series	Moderate to Severe GO	2	P1: 67 P2: 74	1/1	Caucasian	0/2	–	Active (7 point CAS >3)	P1: no previous treatment P2: GCs per os	P1: 5/7 P2: 4/7	P1: 2/7 P2: 3/7	–
Silkiss, 2021 (32)	Retrospective Case Series	Moderate to Severe GO	9	–	1/8	Caucasian (6), Asian (3)	3/6	–	Active (7 point CAS >3)	IVMP (7), MTX (4), GCs per os (3), Orbital radiation (5), Decompressive surgery (3), Plasmapheresis (1)	4	1.2	24.6 weeks
Stevens, 2022 (30)	Retrospective Case Series	Severe GO, Sight-Threatening	3	–	0/3	Caucasian (2), Hispanic (1)	3/0	–	Active (10 point CAS >4)	IVMP (2), GCs (1), Orbital radiation (3), Decompressive surgery (2)	6.66	1.33	–

AZA, azathioprine; CAS, clinical activity score; GCs, glucocorticosteroids; GO, Graves' orbitopathy; IVMP, intravenous methylprednisolone pulses; MMF, mycophenolate mofetil; MTX, methotrexate; NS, not stated; P1, Patient 1; P2, Patient 2; RCT, Randomized clinical trial; RTX, rituximab; SD, standard deviation; TCZ, tocilizumab.

TCZ is administered intravenously at a dose of 8 mg/kg every month for four cycles. As shown in **Table 1**, an average CAS reduction of approximately 3.82 points (CI 95% 2.82-4.83, SD 1.58) was observed across studies after TCZ treatment, achieving a score, declarative of inactive disease in all studies (CAS <3) (20–34). Notably, Perez-Moreiras et al. conducted the only randomized, double-blind and placebo-controlled clinical trial (RCT) with 32 patients, demonstrating a significant decrease in CAS in the TCZ compared to the placebo group (20). Furthermore, they showed an improvement in disease severity and in the Quality of Life (QoL) as assessed by the EUGOGO GO-QoL Questionnaire and the 36-Item Short Form Survey (SF-36). Additionally, TCZ was shown to exert a remarkable effect on each component of CAS, resulting in overall improvements of soft tissue congestive symptoms (20, 24). An important reduction of TSI titer has been also demonstrated in several studies (22, 24–28, 30–34), supporting the evidence that TCZ may have a disease-modifying effect. In a recent retrospective case-control study, 37 patients with moderate-to-severe GO treated with teprotumumab or TCZ were evaluated regarding their clinical response in terms of disease activity and severity, proptosis and diplopia, at weeks 12, 24 and 52 after the first injection of each drug (23). At week 24, all patients in TCZ group demonstrated disease inactivation, 75% of them returned to mild disease and diplopia was improved in 16,7% of patients. However, between weeks 24 and 52, there was a tendency towards disease reactivation and deterioration of disease severity in both TCZ and teprotumumab groups. These findings raise concern about the long-term efficacy of these agents.

**Table 2** summarizes the outcome of TCZ administration on other parameters of GO apart from CAS. Perez-Moreiras et al., in a retrospective longitudinal study including 54 patients with a 22-month follow-up period after TCZ administration, managed to demonstrate a reduction in proptosis and eyelid retraction, leading to diplopia and extraocular motility improvement, along with a post-treatment normalization of the visual field test (22). Regarding proptosis, several other studies reported a diminution of exophthalmos as attested by a reduction of at least 2mm in proptosis measured by the Hertel exophthalmometer (21, 22, 24, 26–30, 32–34). This constitutes a major advantage considering the burden of exophthalmos in eye health and in the patients’ QoL. Nevertheless, not all studies managed to reach statistical significance regarding proptosis amelioration. Interestingly, in the only RCT, no considerable decrease in proptosis was observed in patients receiving TCZ at the 40-week follow-up evaluation (20). Similarly, Tobon et al. did not find any clinically significant outcome in the TCZ group (23). Diplopia is another crucial component of GO as it affects the vision and thus patients’ QoL and safety. The results of the effect of TCZ on diplopia are also controversial as there are studies demonstrating an improvement (22, 23, 25, 26, 28, 32, 33), while others failed to confirm this association or reach statistical significance (20, 24, 34). A possible explanation for these discrepancies may be the chronicity of lesions, implying that long-standing inflammation may lead to irreversible fibrosis. Moreover, extraocular motility is restricted in some patients with GO, because of the enlargement of muscles controlling eye movements, especially the inferior, medial, superior and lateral rectus, resulting in restriction of depression, adduction, elevation and abduction of the eye, respectively (35, 36). To this end, one study managed to show a statistically significant improvement of extraocular motility after TCZ treatment, for the upward and the adduction movements (22). Furthermore, four studies managed to show an increase in visual acuity (21, 22, 26, 28) and three of them additionally reported a decrease in intraocular pressure (21, 26, 28).

**Table 2:** Effect of tocilizumab administration on proptosis, diplopia, visual acuity, visual fields, TSI levels and intraocular pressure.

Author, year	Proptosis	Diplopia	Visual Acuity	Visual Fields	TSI^e^ Levels	IOP^c^
Perrez-Moreiras, 2018 (20)	↔	↔	NA^d^	NA^d^	↔	NA^d^
Sanchez-Bilbao 2020 (21)	↓	NA^d^	↑	NA^d^	NA^d^	↓
Perrez-Moreiras,2021 (22)	↓	↓	↑	↑	↓	NA^d^
Tobon, 2023 (23)	↔	↓	NA^d^	NA^d^	NA^d^	NA^d^
Boutzios, 2023 (24)	↓	↔	NA^d^	NA^d^	↓	NA^d^
Moi, 2021	↓	↓	NA^d^	NA^d^	↓^a^	NA^d^
Lee, 2024 (25)	↔	↓	NA^d^	NA^d^	↓	NA^d^
Bennedjaï, 2020	↓^a^	↓ ^a^	↔	NA^d^	↓^a^	NA^d^
Pampín-Sánchez, 2023 (26)	↓^b^	↓^b^	↑^b^	NA^d^	↓^b^	↓
Jose C-MJ, 2020 (27)	↓^a^	NA^d^	NA^d^	NA^d^	↓	NA^d^
Smith, 2022	NA^d^	NA^d^	NA^d^	NA^d^	↓	NA^d^
Wang, 2024 (28)	↓	↓	↑	NA^d^	↓	↓
Copperman, 2019 (29)	↓^b^	NA^d^	NA^d^	NA^d^	NA^d^	NA^d^
Silkiss, 2020	↓^b^	↓^b^	↑^b^	NA^d^	↓^b^	NA^d^
Stevens, 2022 (30)	↓^b^	NA^d^	NA^d^	NA^d^	↓^b^	NA^d^

↓: decrease, ↑: increase, ↔: no change, ^a^Not statistically significant, ^b^No statistical analysis available, ^c^IOP, intraocular pressure; ^d^NA, not assessed; ^e^Thyroid stimulating immunoglobulin.

Concerning the adverse effects of TCZ implementation, as seen in **Table 3**, hypercholesterolemia was remarked in seven of the studies included in this review, but it was mainly transient or efficiently controlled with oral antilipemic therapy (20, 22, 24, 26, 30, 31, 33). Neutropenia that ranged from mild to severe, was also reported in a small percentage of patients in five of the studies (20–22, 24, 26, 33, 34). Other less common adverse reactions included elevation in liver enzymes (20, 22, 34), mild infections of the upper respiratory tract (20–22, 28, 33), thrombocytopenia (22), skin reactions, such as cutaneous rash, pruritus, urticaria, dermatitis, cellulitis and delayed skin reaction in the site of injection for the subcutaneous form (22, 25, 28, 33, 34), asthenia/fatigue (22, 30), increase in body weight (24), acute pyelonephritis (20), acute pancreatitis (28) and recurrence of herpes zoster (25). Finally, one patient in the Perrez-Moreiras et al. retrospective study developed anaphylactic shock with bronchospasm during the administration of IV TCZ (22). There were no deaths attributed to TCZ administration in the included studies.

**Table 3:** Data from previous studies regarding adverse effects after tocilizumab administration.

Author, year	Hyper-cholesterolemia	Skin reactions	Neutropenia	Transaminases elevation	Upper respiratory tract infection	Body weight increase	Acute pyelonephritis	Acute pancreatitis	Asthenia, fatigue	Headache	Herpes zoster reactivation	Anaphylactic shock
Perrez-Moreiras, 2018 (20)	+		+	+	+		+			+		
Sanchez-Bilbao 2020 (21)			+		+							
Perrez-Moreiras,2021 (22)	+	+	+	+	+				+			+
Tobon, 2023 (23)	No adverse reactions were observed in the tocilizumab group											
Boutzios, 2023 (24)	+					+						
Moi, 2021	+	+	+		+							
Lee, 2024 (25)		+								+	+	
Bennedjaï, 2020		+	+	+								
Pampín-Sánchez, 2023 (26)	+		+									
Jose C-MJ, 2020 (27)	No adverse reactions were observed after tocilizumab administration											
Smith, 2022	+											
Wang, 2024 (28)		+	+		+			+				
Copperman, 2019 (29)	No adverse reactions were observed after tocilizumab administration											
Silkiss, 2020	No adverse reactions were observed after tocilizumab administration											
Stevens, 2022 (30)	+								+			

+, adverse reaction observed.

## 4 Tocilizumab administration in minors

We found only one study that included minors (≤18 years old) to receive TCZ (28). This was a retrospective observational study conducted in China with a total of 79 patients, of which 15 were pediatric cases, whose guardians refused corticosteroid treatment. After administering TCZ, they managed to show a statistically significant reduction in CAS, proptosis and TSI levels, confirming the drug’s efficacy in this population. Nevertheless, safety issues were raised, as an increase in exophthalmos and a deterioration of visual acuity was observed in the pediatric group during the follow-up period, without however persisting at the end of follow-up.

## 5 Choosing between intravenous and subcutaneous route of administration

The question of choosing between subcutaneous and intravenous route for TCZ administration has been raised by some authors. Three case series studies with a small number of patients were conducted using subcutaneous TCZ (SC-TCZ) and the results are notable (29, 30, 32). Copperman et al. were the first to examine the use of SC-TCZ in two patients and achieved to demonstrate an improvement in CAS, proptosis and patients’ QoL (29). Moreover, Silkiss et al. observed a significant decrease in CAS, stable or dropping TSI levels and an amelioration in exophthalmos, diplopia, extraocular motility, eyelid edema, visual acuity and color vision (32). Stevens et al. also noted an improvement in CAS post-TCZ treatment and a 2mm reduction in proptosis in two of the three patients (30).

SC -TCZ is administered at a dose of 162 mg every week or every other week, depending on the cumulative dosage aimed to achieve. This route of administration may have some potential benefits, including the ability of at home self-administration and easier dosage calculation. Cost is another aspect to consider, given that TCZ is a drug used off-label for GO and the subcutaneous form is more affordable than the intravenous one.

## 6 Discussion

Tocilizumab has recently emerged as an alternative option for steroid-resistant moderate-to-severe cases of active GO. While it is administered off-label, data from currently available studies support that it has a clinically significant effect in disease control by hindering the inflammatory process in the retro-ocular tissues. Several studies have managed to show a positive effect of TCZ in disease inactivation and in TSI levels reduction, while the only RCT additionally demonstrated an improvement in disease severity and patients’ QoL (20). Whether TCZ has the potential for a long-standing impact in reducing proptosis and diplopia constitutes a more controversial question, since studies remain inconclusive (20–34). Given the fact that TCZ targets active inflammation, it is reasonable to assume that its effect would be more prominent in eyes with active and early disease and therefore it is of imperative importance to diagnose moderate-to-severe GO cases as early as possible and refer them to a specializing center. Early and timely therapeutic intervention with TCZ is anticipated to withhold the inflammatory process, modify disease course and avoid progression to inactive phase with fibrotic changes, that may not respond to immunosuppressive therapy. Regarding the choice of administration route, intravenous TCZ has been mainly investigated in studies for GO with its efficacy better established than that of the subcutaneous form.

In the studies mentioned in this review, all patients had previously received glucocorticoids to control ocular inflammation, with poor response or relapse after treatment discontinuation. Other immunosuppressive agents, such as methotrexate, azathioprine and mycophenolate mofetil, had been used, without sufficient clinical response. Orbital radiotherapy, decompressive eye surgery and plasmapheresis were also implemented as depicted in **Table 1**. There was no previous administration of biological agents in patients treated with TCZ, except for one patient in the Ceballos-Macias et al. case series, who had received rituximab (27). Therefore, the heterogeneity of first-line background treatment does not allow us to draw safe conclusions on the optimal therapeutic interventions to achieve the maximum clinical benefit regarding the introduction of TCZ as second-line treatment.

Regarding patients’ smoking status, tobacco use is a well characterized risk factor associated with the progression of GO and thus it constitutes a considerable parameter when examining patients’ characteristics (9, 37–39). Previous studies have demonstrated that smokers with GD experience higher risk of developing severe GO than non-smokers, while patients with GO are more likely to be smokers than those without (37–39). Moreover, smoking may delay the response to treatment, while tobacco cessation enhances the clinical outcome of GO therapy (9, 37–39) highlighting the importance of quitting smoking as a preventive measure before starting TCZ treatment. Smoking status in all previously mentioned studies has not been considered adequately and thus TZC efficacy is at least in part subject to bias.

Another important risk factor that is involved in the development of GO is lipid profile. Studies have proved an association between high serum levels of total cholesterol and low-density lipoprotein (LDL) and the presence of GO (40–42). Notably, Stein et al. managed to show that statin use lowers the risk of GO development in patients with recent onset GD (43). Additionally, Lanzolla et al. performed an RCT in which 2 groups of patients with moderate-to-severe GO were administered intravenous glucocorticoids (ivGCs) for twelve weeks with or without the addition of atorvastatin (20 mg once daily) for twenty-four weeks. Patients who received atorvastatin in combination with the ivGCs displayed a superior response compared to the ivGCs only group (44). Finally, Nilsson et al. carried out a large register-based study in Sweden and found that statins provided a statistically significant protection against the occurrence of GO in patients with GD (45). This outcome has not been established with other lipid-lowering agents but only with statins, and especially atorvastatin, supporting the notion that the anti-inflammatory rather than the lipid-lowering effect mediates this protective action (43, 45). Thus, adequate control of cholesterol levels is a prerequisite to increase the efficacy of TCZ treatment. In the studies mentioned previously, there is no information about the lipid profile which may have influenced the net effect of TCZ.

It is worth mentioning that approximately one third of children with juvenile GD are affected by GO and the first-line treatment of moderate-to-severe cases consists of corticosteroids. Nevertheless, efficacy and safety of corticosteroids in this population are questionable. In the only study that explored the administration of TCZ in pediatric patients the results were promising but not without safety issues to consider (28).

Regarding prediction of corticosteroid treatment response in patients with active moderate-to-severe GO, microRNAs have recently emerged as possible predictive biomarkers. miRNAs are molecules that control inflammation through modulation of molecular pathways, such as the NF-κB/NLRP3 pathway and have been implemented in the pathogenesis of GO (46). Of note, Shen et al. found a positive predictive value of lower serum miR-224-5p and corticosteroid resistance (47), while Manso et al. showed an association between higher levels of circulating miR-146a in the patients’ serum and response to corticosteroids (48). Measuring these molecules before treatment initiation can reduce corticosteroid-administration-associated adverse effects while improving patient care with the implementation of precision medicine resources.

Although the outcomes of the studies mentioned in this article are encouraging regarding TCZ safety and efficacy for moderate-to-severe GO cases, there are some limitations to be considered. The most important is the small number of patients and the relatively homogenous patient sample across studies, that limit study precision and generalizability. Moreover, most of the studies were retrospective and observational and thus no control arm was available, limiting the study quality and the level of evidence. What may also be considered a limitation is the short follow-up time that did not allow long-term effects and potential safety issues to be thoroughly explored. In many studies different treatment regimens were previously or concurrently administered and the time between their cessation and the initiation of TCZ was heterogenous, leading to the assumption that beneficial results cannot be safely attributed only to TCZ. Similarly, smoking status and lipid profile have not been adequately considered or adjusted appropriately to evaluate the net effect of TCZ.

In conclusion, despite the low level of evidence, TCZ presents a beneficial efficacy and safety profile with potential to limit inflammation and improve disease activity and severity in patients with active moderate-to-severe steroid-resistant GO. Nevertheless, randomized controlled clinical trials, with larger number of patients and longer follow-up period, which will consider first line treatment and various confounders, are required to provide more solid evidence concerning the effectiveness of TCZ.
